# MicroRNA-449a Is Downregulated in Non-Small Cell Lung Cancer and Inhibits Migration and Invasion by Targeting c-Met

**DOI:** 10.1371/journal.pone.0064759

**Published:** 2013-05-29

**Authors:** Wenting Luo, Bo Huang, Zixuan Li, Haiying Li, Limei Sun, Qingfu Zhang, Xueshan Qiu, Enhua Wang

**Affiliations:** 1 Department of Pathology, First Affiliated Hospital and College of Basic Medical Sciences, China Medical University, Shenyang, China; 2 Department of Pathology, Liaoning Tumor Hospital, Shenyang, China; H.Lee Moffitt Cancer Center & Research Institute, United States of America

## Abstract

MicroRNA-449a is expressed at a low level in several tumors and cancer cell lines, and induces G1 arrest, apoptosis, and senescence. To identify the function of miR-449a in non-small cell lung cancer (NSCLC), we discussed the potential relevance of miR-449a to clinicopathological characteristics and prognosis in NSCLC. We also investigated the impact of miR-449a on migration and invasion in NSCLC cells. The expression of miR-449a in NSCLC tissues and cell lines was detected using RT-qPCR. *In vitro*, gain-of-function, loss-of-function experiments, and fluorescence assays were performed to identify the potential target of miR-449a and the function of miR-449a in NSCLC cells. MiR-449a was downregulated in both NSCLC tissues and cell lines. Moreover, a low expression level of miR-449a appeared to be correlated with lymph node metastasis and poor survival. *In vitro*, miR-449 regulated cell migration and invasion in NSCLC cells as a potential tumor suppressor, at least in part by targeting c-Met. Furthermore, reciprocal expression of miR-449a and c-Met was shown in NSCLC tissue samples. This study indicates that miR-449a might be associated with NSCLC progression, and suggests a crucial role for miR-449a in NSCLC.

## Introduction

MicroRNAs (miRNAs) are a class of small non-coding RNAs, approximately 20 to 25 nucleotides, which regulate gene expression posttranscriptionally. By binding to a complementary sequence predominantly found in the 3**′**-UTR of target mRNAs, miRNAs either degrade these mRNAs or inhibit them from being translated into proteins. Nearly 50% of human miRNAs are located at fragile sites and genomic regions involved in cancers [1]. Emerging evidence shows that miRNAs are correlated with various human cancers and function as both oncogenes and tumor suppressors [2], [3], [4].

MicroRNA expression deregulation in human cancers have shown that miRNA dysregulation is associated with many cancers including lung cancer [5], [6], [7], [8], [9]. High expression of miR-155 and low expression of let-7, miR-126 are reported to predict poor prognosis of lung adenocarcinoma [6], [7], [10]. Raponi and colleagues described distinct miRNA expressions in lung squamous cell carcinoma (SCC) compared with normal lung tissue [11]. Based on miRNA expression deregulation, miRNAs could provide a novel method for cancer diagnosis.

MiR-449 is expressed at a low level in several cancer cell lines and solid tumors including prostate cancers [12], gastric cancers [13], bladder cancers [14] and lung cancer [15]. The biological targets of miR-449 have been partially identified, and miR-449 induces G1 arrest, apoptosis, and senescence by regulation of key factors in cell cycle and apoptosis such as histone deacetylase 1 (HDAC1) [12], CDK6 [16], [17], [18], CDC25A [16], [18], cyclin D1(CCND1) [19], and SIRT1 [17]. However, little is known about the role of miR-449a in NSCLC progression. In this study, we found that miR-449a was downregulated in NSCLC tissues and associated with clinicopathologic characters and prognosis. We explored the effect of miR-449a in NSCLC cell migration and invasion and its regulation of the target gene c-Met. Reciprocal expression of miR-449a and c-Met was observed in NSCLC tissue samples and the possible roles of miR-449a and c-Met in NSCLC progression are discussed.

## Materials and Methods

### Samples

Fresh samples from lung cancer and corresponding normal adjacent tissue (NAT, >5 cm from the cancer tissue) were obtained from patients at First Affiliated Hospital of China Medical University and Liaoning Cancer Hospital between January 2008 and November 2009 with informed consent. (The hospital’s Ethical Review Committee gave approval). None of the 84 patients in the study received any chemotherapy or radiation therapy before surgery. Ten normal lung specimens were obtained from consenting patients during surgery for benign lung disease. For miR-449 quantitative analysis, formalin-fixed paraffin-embedded tissues (FFPETs) from lung cancer cases were selected randomly from patients at Liaoning Cancer Hospital January 2004 to December 2005 with informed consent and approval. Clinicopathologic information was available and two pathologists independently determined diagnoses and histological grade based on World Health Organization guidelines. Clinicopathologic information and follow-up data are summarized in Table S1.

This study was conducted with the approval of the local institutional review board at First Affiliated Hospital of China Medical University and Liaoning Cancer Hospital. Written informed consent was obtained from all patients and all clinical investigation have been conducted.

### Real-time Quantitative Polymerase Chain Reaction (RT-qPCR) of miR-449a

Total RNA was extracted from fresh tissues and cells using Trizol (Invitrogen, NY, USA) according to the manufacturer’s instructions. RNA from FFPETs was separated using an miRNA Isolation Kit Am1975 RecoverAllTM (Ambion, Austin, USA) according to the manufacturer’s instructions. MicroRNA quantification from extracted RNA was performed using TaqMan MicroRNA Assays (Applied Biosystems, Foster City, USA). RT primer and TaqMan probe of miR-449a (Applied Biosystems, Foster City, USA) were used for PCR analysis on an ABI 7900HT (Applied Biosystems, Foster City, USA) in accordance with the manufacturer’s protocol. RNU6B, as an internal control, was performed in the same manner as above. Each RT-qPCR analysis was performed in three independent experiments, using three independent samples. The relative quantity of miR-449a in tissues and NSCLC cell lines were compared to the mean expression of 10 normal samples, using the equation RQ = 2^–ΔΔCT^ [20], [21].

### Cell Culture and Transfection

The human lung cancer cell line A549 and H1299 cell lines were obtained from American Type Culture Collection. Other NSCLC cell lines BE1 and LH7 cells were from previous published paper of our team [8]. A549 were grown in Dulbecco’s Modifed Eagle Medium (Gibco® Invitrogen, Carlsbad, USA). BE-1, LH-7 and H1299 were grown in RPMI-1640 (Gibco® Invitrogen, Carlsbad, USA). In each case, medium was supplemented with 10% fetal bovine serum (Hyclone, Logan, USA), 100 U/ml penicillin, and 100 U/ml streptomycin. Cells were incubated at 37°C in a humidified incubator containing 5% CO_ 2_.

A mimic negative control, an miR-449a mimic, an inhibitor negative control and an miR-449a inhibitor, were from RiboBio (Guangzhou, China). A549 and H1299 cells were transfected using HiPerFect Transfection Reagent (Qiagen, Hilden, German). Briefly, complexes containing the mimics or inhibitors were prepared according to the manufacturer’s protocol. The mixture was added to cells at a final concentration of 100 nM. The four groups were: cells transfected with a mimic negative control, transfected with an miR-449a mimic, transfected with an inhibitor control, transfected with an miR-449a inhibitor. In addition, a c-Met small interfering RNA (siRNA) and a negative control were designed and synthesized by GenePharma (Shanghai, China) and transfected into A549 cells. The sequences of the c-Met siRNA were 5**′**-GUC AUA GGA AGA GGG CAU UTT-3**′** (sense), and 5**′**-AAU GCC CUC UUC CUA UGA CTT-3**′** (antisense).

### Cell Migration and Invasion Assays

Transwell chambers (Corning, NY, USA) with a pore size of 8 µm were used for cell migration and invasion assays. Cells were grown to 60% confluency and transfected with the miRNA or siRNA. After 24 hours, for migration assay, cells were trypsinized and 5×10^4^ cells were resuspended in serum-free medium and placed in the upper chamber. As a chemoattractant, the lower chamber contained 10% FBS. Cells were incubated at 37°C in 5% CO_ 2_ for 16 hours, and nonmigrating cells were removed with a cotton swab. Migrated cells washed twice with PBS and fixed in 100% methanol, stained with haematoxylin. Stained cells were viewed under a microscope (×200 Magnification), and the number of migrated cells was counted in five random fields. For invasion assays, the upper chamber was precoated with Matrigel mixed with serum-free medium (diluted at 1∶3, BD Biosciences, San Jose, USA). After solidification of the mixture, 5×10^4^ cells in serum-free medium were placed into the upper chamber. The lower chamber contained 10% FBS as a chemoattractant. Cells were incubated at 37°C in 5% CO_ 2_ for 18 hours, and non invading cells were removed with cotton swab. Invasive cells were fixed, stained and counted. Stained cells were viewed under a microscope (×200 Magnification), and the number of migrated cells was counted in five random fields. Assays were performed in duplicate in three independent experiments.

### RT-qPCR Analysis of miR-449a Target Gene

Total RNA was extracted from cells, and cDNA synthesis was performed with PrimerScript RT reagent kit (Takara, Dalian, China). The resulting cDNA was amplified using c-Met primers with SYBR Premix Ex Taq II (Takara, Dalian, China) with the parameters 95°C for 30 s, followed by 40 cycles of 95°C for 5s, 60°C for 30 s. Primers for c-Met were F: 5**′**-GGCTGGTGGCACTTTACTTA-3**′** and R: 5**′**- CTTGTCTCTCGGTTGGCTA-3**′** , and primers for endogenous β-actin were F: 5**′**-AGCACAGAGCCTCGCCTTTG-3**′** , R: 5**′**-ACATGCCGGAGCCGTTGT-3**′** . Melting curve analysis was carried out at the end of the cycles to ensure product specificity. The relative quantity of c-Met, normalized to β-actin, calculated based on the equation RQ = 2^–ΔΔCT^.

### Western Blot Analysis

Tissue samples of 0.2 g were ground to powder in liquid nitrogen. Tissue powder and cells were extracted with lysis buffer (150 mM NaCl, 1% NP-40, 0.1% SDS, 2 mg/mL aprotinin and 1 mM PMSF) for 30 min at 4°C. An equal amount of protein was separated on 10% SDS-PAGE gels. Anti-c-Met (21220, Signalway Antibody, Maryland, USA), anti-MMP2 (sc-13595, Santa Cruz, CA, USA), anti-MMP9 (sc-13520, Santa Cruz, CA, USA), anti-β-Actin (sc-1616, Santa Cruz, CA, USA) were used followed the manufacturer’s instructions. Image J software (National institutes of Health, Md, USA) was used to measure the intensity of protein bands.

### Fluorescent Reporter Assay

In order to perform the fluorescent reporter assay, the following primers were used to amplify the 3**′**UTR of the c-Met gene from human cDNA (NM_000245): forward primer 5′-GATCCTGCTAGTACTATGTCAAAGCAACAGTCCACACTTTGTCCAATGGTTTTTTCACTGCCTGAG -3**′** , reverse primer 5**′**-AATTCTCAGGCAGTGAAAAAACCATTGGACAAAGTGTGGACTGTTGCTTTGACATAGTACTAGCAG-3**′** .

The plasmid containing c-Met 3**′**UTR to a fluorescent reporter was constructed (Sauer Biotechnology Inc, China). A549 cells were seeded into 48-well plates, cultured overnight, then cotransfected with mimic control, miR-449a mimic, inhibitor control, miR-449a inhibitor followed by the pcDNA3/EGFP-c-Met 3**′**UTR reporter vector after 24 hours. The enhanced green fluorescent protein (EGFP) activity was normalized to red fluorescent protein (RFP) activity. After 72 hours the proteins were extracted, and the Fluorescence Spectrophotometer F-4500 (HITACHI, Japan) was used to determine the fluorescence intensity. Assays were performed in duplicate in three independent experiments.

### Statistical Analysis

SPSS13.0 statistical software package (SPSS, Chicago, USA) was used for statistical analysis. For RT-qPCR, the expression level of miR-449a in lung cancer and paired NATs was log2 transformed. All values were reported as mean with standard deviation (SD). A paired-samples T-test was used to analyze differences in miR-449a expression between lung cancer and matched NATs. A chi-square test was used to analyze the relationship between miR-449a expression levels and clinicopathologic characters. The Mann-Whitney U test was used to analyze the ranked data of histological grade and pathological stage. Spearman determined the correlation between miR-449a expression and pathological stage, and lymph node status. The Kaplan-Meier method was used for survival curves, and a log-rank test was used for comparison. Cox regression was used to examine the effect of covariables. The results of cell experiments were analyzed by an independent samples T-test and one-way ANOVA. *P*<0.05 was considered statistically significant.

## Results

### 1. MiR-449a was Downregulated in NSCLC Tissues and Associated with Lymph Node Metastasis

**Figure 1:**
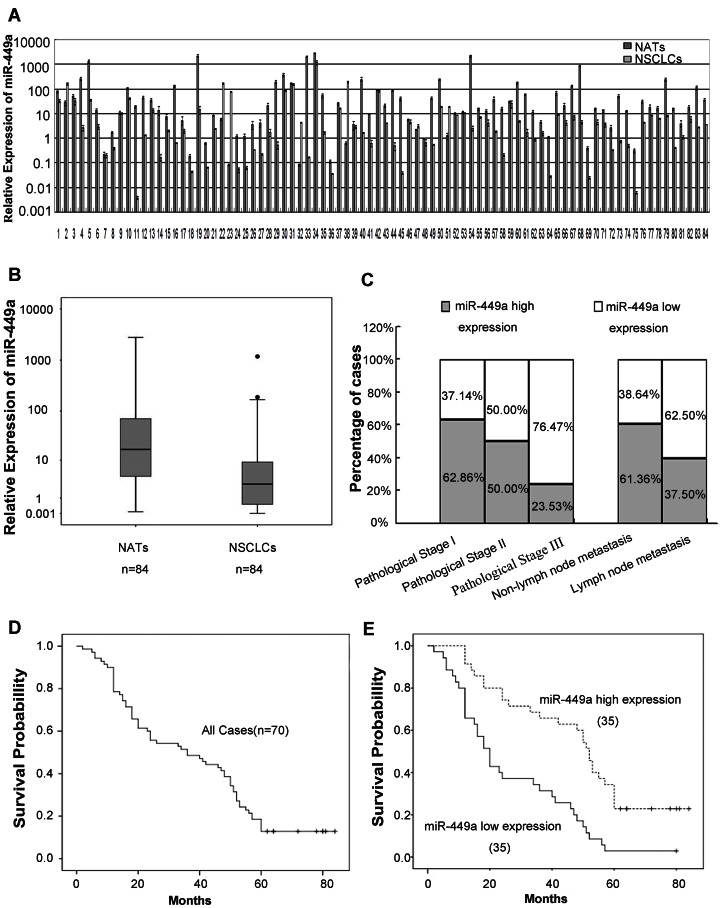
Low expression level of miR-449a was correlated with poor prognosis of lung cancer patients. A. Paired bar chart indicates downregulation of miR-449a in NSCLC tissues. RT-qPCR result of miR-449a in 84 fresh NSCLC tissues and paired NATs. Triplicate experiments were completed for each sample, the relative expression was calculated using the equation RQ = 2^–ΔΔCT^. Compared with NATs, miR-449a was significantly downregulated in NSCLC samples (paired T-test, *P = *0.004, *t* = −2.997). B. Boxplot indicates downregulation of miR-449a in NSCLC tissues. C. Correlation between miR-449a expression level and pathological stage, lymph node status of 84 fresh paired samples from lung cancer patients. D. Kaplan-Meier curve for 5-year overall survival rates (14.29%) of 70 FFPET samples from lung cancer patients. E. In 70 FFPET samples from lung cancer patients, Kaplan-Meier curve for lung cancer patients classified as high or low miR-449a expression, low expression level of miR-449a was significantly correlated with patient poor survival (log-rank test, *P*<0.001).

We detected expression of miR-449a by RT-qPCR in 84 fresh NSCLC tissue samples with paired NATs. Compared with NATs, the expression level of miR-449a (77/84) was significantly downregulated in NSCLC tissues (paired T-test, *P = *0.004, *t* = −2.997, Figure 1A). Downregulation of miR-449a detected by RT-qPCR in NSCLC tissues, was also shown in the boxplot (Figure 1B). RNU6B was used as an internal standard.

To determine the effects of miR-449a expression on tumor initiation and progression, NSCLC patients were divided into two groups, low and high expression, according to the mean expression level of miR-449a in cancerous tissues (log2 value, median = 1.47). Correlation between miR-449a expression and clinicopathologic variables of lung cancer are shown in Table 1. Significant correlations were observed between miR-449a expression and pathological stage (*P* = 0.012) (Mann-Whitney U test). In 17 cases presenting with pathological stage III, 13 (76.47%) cases had low expression of miR-449a, while the low expression rate was 16/32 (50.00%) of pathological stageII and 13/35 (37.14%) of pathological stage I (Figure 1C). Changes in miR-449a expression were also significantly associated with lymph node metastasis. In 40 cases of lung cancer with lymph node metastasis, 25 (62.5%) had low expression of miR-449a. By contrast, in 44 cases without lymph node metastasis, only 17 cases (38.64%) had low miR-449a expression (*P* = 0.029, chi-square test; Figure 1C). No correlation was observed between miR-449a expression and gender, age, histology type or histological grade.

**Table 1:** Relationship between miR-449a expression and clinicopathologic factors in 84 fresh samples of lung cancer patients.

	miR-449a			
Variable	Patients	Low expression	High Expression	*P* value
Age(years)				
≥60	45	26(57.8%)	19(42.2%)	0.126^a^
<60	39	16 (41.0%)	23(59.0%)	
Gender				
Male	59	31(52.5%)	28(47.5%)	0.474^a^
Female	25	11(44.0%)	14(56.0%)	
Size				
>3 cm	50	23(46.0%)	27(54.0%)	0.374^a^
≤3 cm	34	19(55.9%)	15(44.1%)	
Location				
Right	43	23(53.5%)	20(46.5%)	0.513^a^
Left	41	19(46.3%)	22(53.7%)	
Histology type				
Adenocarcinoma	43	19(44.2%)	24(55.8%)	0.275^a^
Squamous cancer	41	23(56.1%)	18(43.9%)	
Histological grade				0.197^b^
I	30	14(46.7%)	16 (53.3%)	
II	26	10(38.5%)	16(61.5%)	
III	28	18 (64.3%)	10(35.7%)	
Pathological stage				
I	35	13(37.1%)	22(62.9%)	0.012^b^
II	32	16(50.0%)	16(50.0%)	
III	17	13(76.5%)	4(23.5%)	
Lymph node status				
No Metastasis	44	17(38.6)	27(61.4)	0.029^a^
Metastasis	40	25(62.5)	15(37.5)	

a Chi-square Test.

b Mann-whitney Test.

To better understand the correlation between miR-449a expression and pathological stage, lymph node metastasis, the Spearman correlation test was used for further analysis. The results showed a negative correlation between miR-449a expression and pathological stage (r = −0.276, *P* = 0.011), and lymph node metastasis (r = −0.238, *P* = 0.029).

### 2. Correlation between miR-449a Expression and Prognosis of Lung Cancer Patients

MiR-449a expression was detected in FFPETs of 70 lung cancer patients with follow-up data (Table S1). The overall survival curve in Figure 1D shows a 5-year overall survival rate of 14.29% for 70 cases. According to Kaplan-Meier survival analysis, NSCLC patients with low miR-449a expression had a significantly poorer prognosis than those with high miR-449a expression (log-rank test, *P*<0.001; Figure 1E). In addition to miR-449a expression, other clinicopathologic factors such as histological grade (log-rank test, *P* = 0.004), pathological stage (log-rank test, P<0.001) and lymph node metastasis (log-rank test, *P*<0.001) were also associated with prognosis of lung cancer patients according to the survival analysis. Univariate Cox proportional hazard regression analysis was performed to find the most strongly predictive factor for poor prognosis of patients with lung cancer. We found that miR-449a expression (*P*<0.001, hazard ratio [HR] = 0.345), histological grade (*P* = 0.002, HR = 1.725), pathological stage (*P*<0.001, HR = 3.254), and lymph node metastasis (*P*<0.001, HR = 6.535) were predictive factors for poor prognosis of patients with lung cancer (Table 2).

**Table 2:** Relationship between survival time and miR-449a expression level of FFPETs from 70 lung cancer patients.

Variable	Subset	Hazard ratio (95%CI)	*P* value
Univariate analysis(n = 70)			
Age	≥59 versus <59	0.889(0.536–1.473)	0.647
Gender	male versus female	0.970(0.575–1.638)	0.911
Location	right versus left	1.166(0.700–1.941)	0.556
Size	≥3 cm versus <3 cm	1.279(0.766–2.133)	0.347
Histology type	AC versus SCC	0.898(0.543–1.485)	0.675
Histological grade	I VS II VS III	1.725(1.228–2.425)	0.002
Pathological stage	I VS II VS III	3.254(2.166–4.887)	<0.001
Lympho nodes metastasis	“− versus +”	6.535(3.619–11.799)	<0.001
MiR-449a level	low versus high	0.345(0.203–0.587)	<0.001
Multivariate analysis(n = 70)			
Histological grade	I VS II VS III	1.282(0.892–1.844)	0.180
Pathological stage	I VS II VS III	2.162(1.338–3.491)	0.002
Lympho node metastasis	“− versus +”	3.104(1.615–5.966)	0.001
MiR-449a level	low versus high	0.558(0.319–0.976)	0.041

Multivariate Cox proportional hazard regression analysis showed that the expression level of miR-449a (*P* = 0.041, HR = 0.558, 95% confidence interval [CI]: 0.319–0.976) was an important favorable prognostic factor independent of other clinicopathologic factors, for instance pathological stage (*P* = 0.002, HR = 2.162, 95% CI: 1.338–3.491), lymph node status (*P* = 0.001, HR = 3.104, 95% CI: 1.615–5.966; Table 2).

### 3. MiR-449a was Downregulated in NSCLC Cell Lines and Affected Migration and Invasion *in vitro*

**Figure 2:**
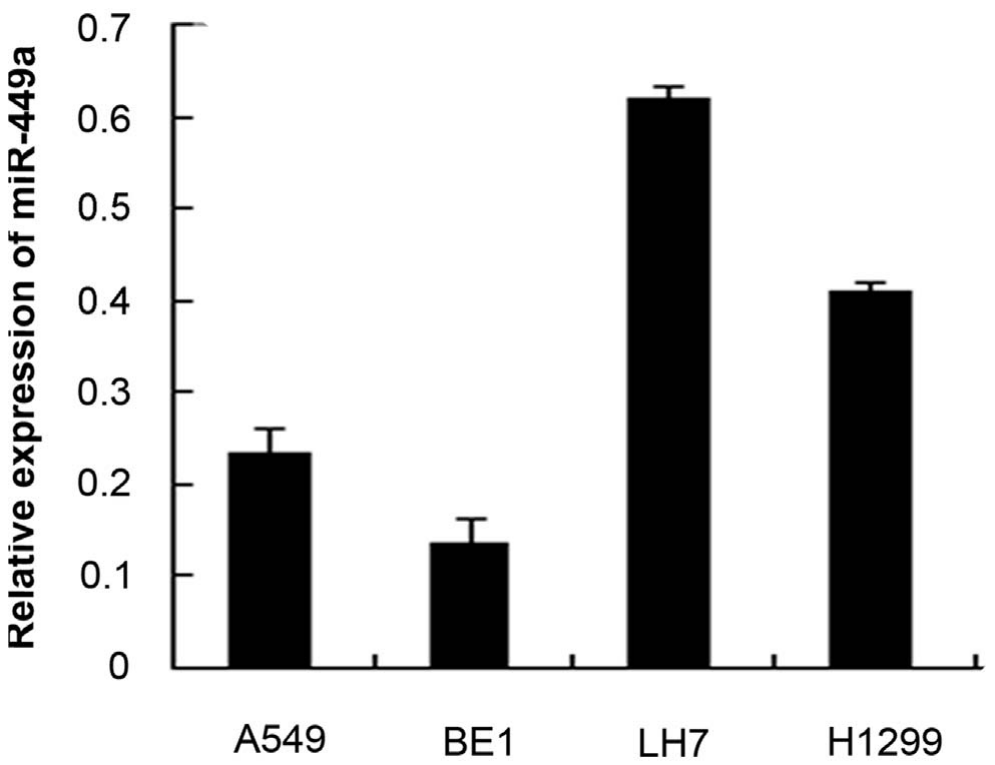
Expression of miR-449a was downregulated in NSCLC cell lines. The relative quantity of miR-449a in 4 NSCLC cell lines were compared to the mean expression level of 10 normal lung specimens based on the equation RQ = 2^–ΔΔCT^.

We examined the expression of miR-449a in 4 NSCLC cell lines: A549, BE-1, LH-7, H1299 using RT-qPCR. As shown in Figure 2, compared to the mean expression of 10 normal lung specimens, all 4 cell lines showed significant downregulation of miR-449a. Additionally, expression of miR-449a was found to be considerably decreased in the highly invasive NSCLC cell line BE1 compared with the less invasive LH7 cell line.

MiR-449a expression in both tissues and NSCLC cell lines suggested that miR-449a was associated with metastasis. Therefore, we explored the potential effects of miR-449a on migration and invasion in lung cancer cells. According to the expression of miR-449a in NSCLC cell lines, we selected A549 and H1299 cells lines, which had relatively moderate expression of miR-449a, for transwell assays. To determine the effect of miR-449a on cell migration and invasion, A549 and H1299 cells were transfected with mimic control, miR-449a mimic, inhibitor control and miR-449a inhibitor. To ensure transfection effects, RT-qPCR was performed to identify expression of miR-449a after 24 hours (Figure S1). The relative expression of miR-449a in A549 and H1299 cells transfected with a mimic was significantly increased.

**Figure 3:**
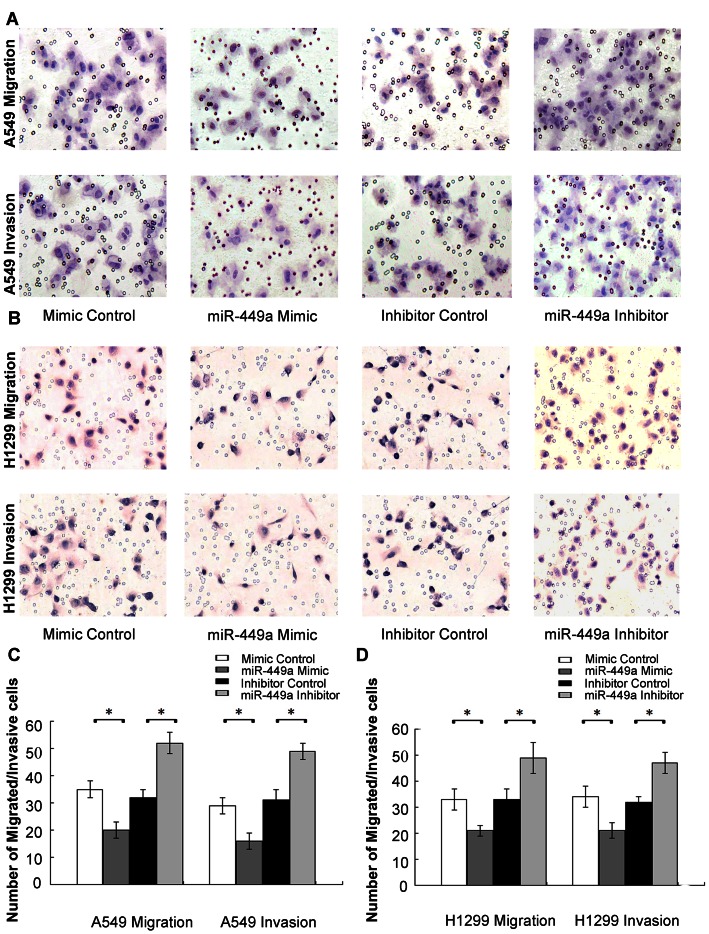
Effects of miR-449a on migration and invasion in NSCLC cells. A&B. MiR-449a regulated cell migration and invasion. A549/H1299 cells were transfected with Mimic Control, miR-449a Mimic, Inhibitor Control and miR-449a Inhibitor, then subjected to migration and invasion assays as described in Section 2. Migration/invasive cells were imaged for representative photographs after staining with Haematoxylin. Original Magnification, ×200. C&D. Average migrated/invasive cell number from three independent experiments was shown in bar chart, mean±SD. *P<0.01.

As shown in Figure 3, compared with controls, the migratory capabilities of cells transfected with the miR-449a mimic were reduced by approximately 42.9% for A549 cells and 36.4% for H1299 cells. By contrast, cells in the miR-449a inhibitor group had higher migration ability than controls, the migrated cells increased by 62.5% for A549 and 48.5% for H1299 cells (Figure 3). Matrigel invasion assays were also performed, and exogenously increase of miR-449 expression reduced the number of invasive cells significantly by 44.8% for A549 and 38.2% for H1299 cells (Figure 3). The number of invasive cells increased significantly by 58.1% for A549 and 46.9% for H1299 cells when transfected with miR-449a inhibitor (Figure 3).

### 4. Mir-449a Targeted c-Met

**Figure 4:**
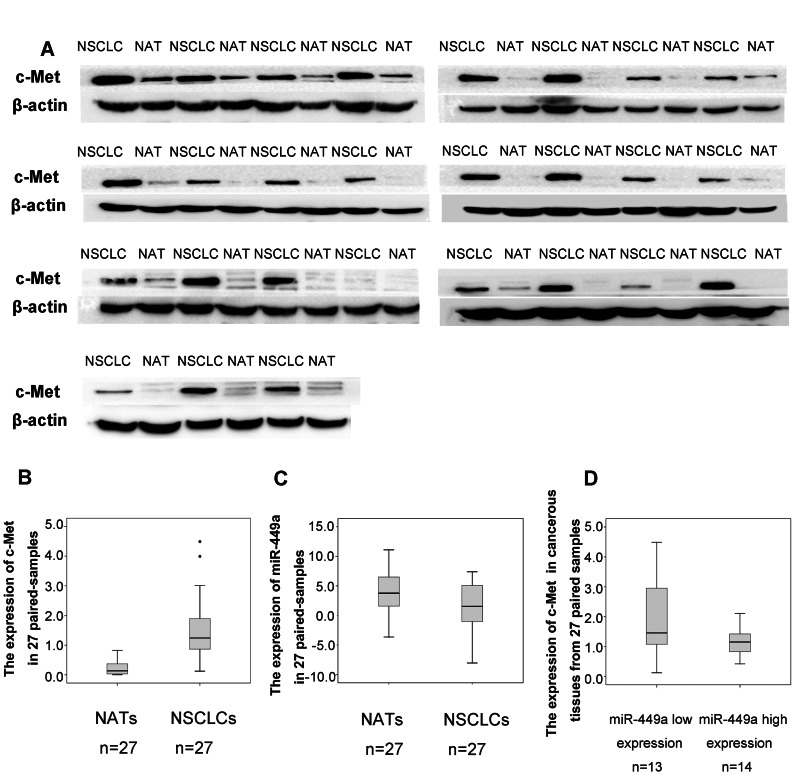
Expression of miR-449a was reciprocally correlated with c-Met in NSCLC tissues. A&B. Expression of c-Met detected by Western Blot in 27 pairs of fresh NSCLC samples. β-actin was used as an internal control. The intensity of protein bands were measured, the y-axis of boxplot was based on OD. Values of Western Blot. C. Relative expression of miR-449a in the same panel of 27 paired NSCLC samples. D. Expression of c-Met was shown grouped by the mean expression level of miR-449a in 84 cancerous samples tissues. The expression of c-Met in miR-449a low expressers was higher than miR-449a high expressers. The intensity of protein bands were measured, the y-axis of boxplot was based on OD. Values of Western Blot (*P* = 0.037).

To determine possible target genes of miR-449a in NSCLC cells, we used three algorithms to analyze potential targets of miR-449a: TargetScan 5.2 (June 2011), miRanda (August 2010), and Diana-microT (July 2011). In the potential target list, c-Met which has two predicted binding sites with miR-449a is implicated as a key mediator of cell migration, invasion, and metastasis in tumorigenesis and tumor progression in a variety of tumors including NSCLC (Figure S2). We examined whether c-Met was regulated by miR-449a in NSCLC tissue samples and *in vitro*. To determine the clinical relevance of miR-449a and c-Met, we selected 27 pairs from the 84 fresh NSCLC tissue samples with paired NATs according to the mean expression level of miR-449a in 84 cancerous samples tissues, including 13 miR-449a low expressers and 14 miR-449a high expressers. As shown in Figure 4 A&B, western blots were performed to examine the expression level of c-Met in these 27 pair samples, c-Met expression was significantly higher in NSCLC tissues compared with NATs (paired T-test, *P*<0.001, *t* = 6.352), while miR-449a was down-regulated in the same panel of NSCLC tissues as detected in Result 1(Figure 4C). Furthermore, tumors with low expression level of miR-449a revealed high concentrations of c-Met, while tumors with high expression level of miR-449a performed low concentrations of c-Met (*P* = 0.037, Figure 4D). These results indicated that an inverse correlation between miR-449a and c-Met expression in tissue samples.

**Figure 5:**
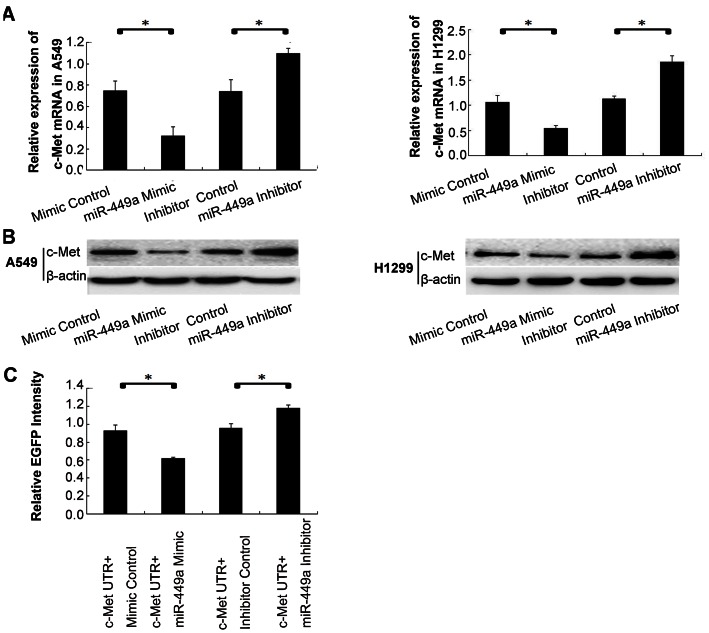
MiR-449a targeted c-Met in NSCLC cells. A. The effect of miR-449a on c-Met mRNA in NSCLC cells. A549/H1299 cells were transfected with a Mimic Control, an miR-449a Mimic, an Inhibitor Control and an miR-449a Inhibitor, then RNA was extracted and subjected to RT-qPCR. The relative quantity of c-Met, normalized to β-actin, were compared to Mock group based on the equation RQ = 2^–ΔΔCT^. *P<0.01. B. The effect of miR-449a on c-Met protein in NSCLC cells. A549/H1299 cells were transfected with a mimic Control, an miR-449a mimic, an Inhibitor Control and an miR-449a Inhibitor. After 48 hours, cellular protein was extracted and subjected to Western Blot analysis of c-Met. β-actin was used as an internal control. C. MiR-449a binding sites in the 3′UTR of c-Met was assessed using fluorescent reporter assays. With a vector containing c-Met 3**′**-UTR, A549 cells were transfected with a mimic Control, an miR-449a mimic, an Inhibitor Control and an miR-449a Inhibitor, then protein was extracted and the fluorescence intensity was determined. *P<0.05.

To determine whether miR-449a targeted c-Met *in vitro*, we transfected A549 cells and H1299 cells with miR-449a mimic or inhibitor and detected the level of c-Met mRNA using RT-qPCR at 48 h post transfection. Overexpression of miR-449a significantly decreased c-Met mRNA compared to controls, whereas inhibition of miR-449a resulted in an increase of c-Met mRNA (Figure 5A). Western blot analysis was performed to test the protein expression of c-Met. The results showed overexpression of miR-449a led to a dramatic decrease in c-Met protein, while reduction of miR-449a remarkably increased c-Met protein (Figure 5B). These results indicated that miR-449a regulated the expression of c-Met at both the mRNA and protein levels.

To determine if c-Met was a direct target of miR-449a, fluorescent reporter assays were performed. The 3**′**-UTR of c-Met with two predicted binding sites for miR-449a was cloned into a fluorescent reporter vector. As shown in Figure 5C, upregulation of miR-449a reduced the intensity of EGFP fluorescence in cells transfected with a vector containing c-Met 3**′**-UTR compared with controls, while in the miR-449a inhibitor group the intensity of EGFP fluorescence increased significantly. These results indicate that miR-449a binds to c-Met 3**′**UTR region directly.

**Figure 6:**
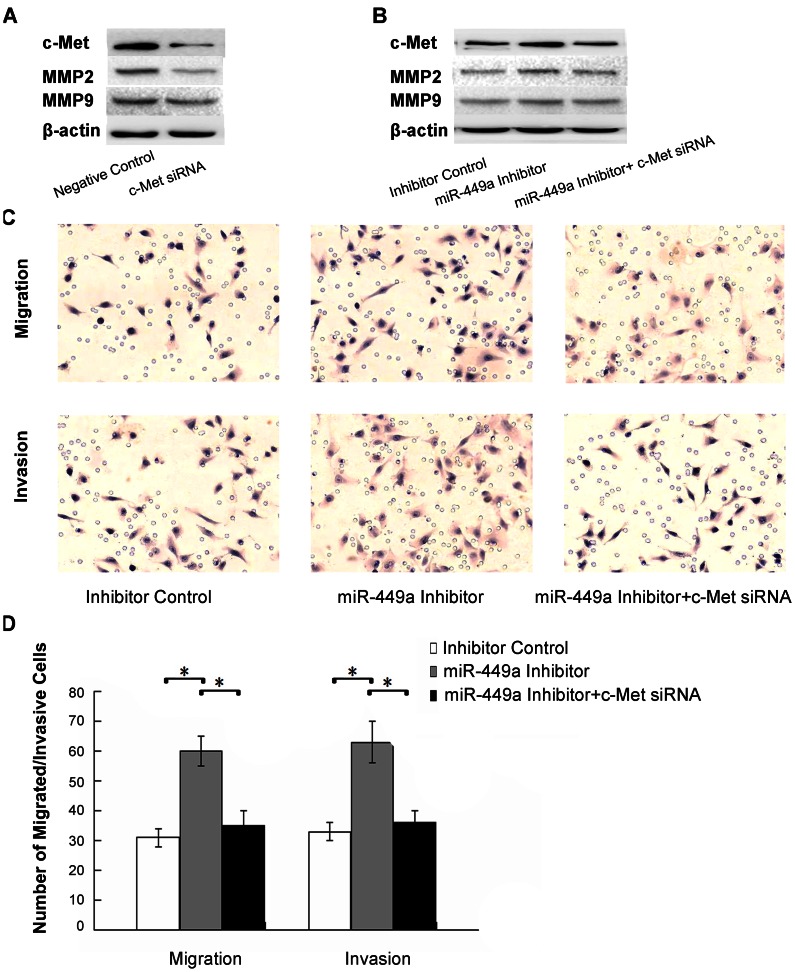
MiR-449a regulated migration and invasion through targeting c-Met. A. C-Met siRNA oligonucleotides were used to silence c-Met expression, and the effect was verified. A549 cells were transfected with a negative Control and a c-Met siRNA, cellular protein was extracted and subjected to Western Blot analysis of c-Met. β-actin was used as an internal control. B. Co-transfection of an miR-449a inhibitor and a c-Met siRNA was performed in A549 cells. Cells were transfected with an inhibitor control, an miR-449a Inhibitor and an miR-449a Inhibitor plus a c-Met siRNA, protein from these cells was extracted and subjected to Western Blot analysis of c-Met. β-actin was used as an internal control. C. The effects of miR-449a on migration and invasion in A549 cells were abolished after co-transfection. A549 cells were transfected with an inhibitor control, an miR-449a Inhibitor and an miR-449a Inhibitor plus a c-Met siRNA, then subjected to migration and invasion assays as described in Section 2. Migration/invasive cells were imaged for representative photographs after staining with Haematoxylin. Original Magnification,×200. D. Average migrated/invasive cell number from three independent experiments was shown in bar chart, mean±SD. *P<0.01.

To further confirm if miR-449a regulated migration and invasion through targeting c-Met, c-Met siRNA was used to silence c-Met expression. Western blot analysis was used to validate the results (Figure 6A). With downstream changes to MMP2 and MMP9 (Figure 6B), co-transfection of miR-449a inhibitor and c-Met siRNA abolished the effects of miR-449a on migration and invasion in A549 cells (Figure 6C&D). These results suggested that miR-449a might be crucial in the regulation of cell migration, invasion, and tumor metastasis through targeting the oncogene c-Met.

## Discussion

We found that miR-449a was significantly downregulated in NSCLC tissues and cell lines, consistent with the report that miR-449 was reduced in several human tumors and cancer cell lines [16]. In addition, Liang and colleagues found that miR-449 was detected in human lung, testis, and fallopian tubes but not other normal human tissues [22], [23]. Moreover, downregulation of miR-449 was observed in gastric cancer. The expression of miR-449 was low or undetectable in 8 of 10 gastric cancer tissues, although no correlation was observed between the reduction in miR-449 expression and clinical characteristics of gastric cancer in the 10 pairs of tissues [13]. Recently, Jeon and colleagues found that miR-449a/b has reduced expression in lung cancer tissues, with the target gene HDAC1 overexpressed at mRNA level [15]. However, data from a large sample set on the expression status of miR-449 and its relevance to clinicopathologic features are unclear. Here, we used 154 NSCLC samples (including 84 fresh samples and 70 FFPET samples) to detect the potential relationship between the expression status of miR-449a and various clinicopathologic characteristics, as well as survival of patients with NSCLC. According to the RT-qPCR results on 84 cases of fresh NSCLC tissues and paired NATs, miR-449a was significantly downregulated in NSCLCs compared with paired NATs. Statistical analysis was performed, and a low level of miR-449a appeared to be correlated with advanced pathological stage, and lymph node metastasis. To our knowledge, our analysis indicated for the first time that miR-449a was associated with lymph node metastasis. Moreover, data from 70 FFPETs indicated that lung cancer patients with low miR-449a expression had poor prognosis. Multivariate Cox regression analysis showed that low expression level of miR-449a was independently associated with worse prognosis as well as advanced pathological stage and lymph node metastases. These results suggested that miR-449a might be a useful prognostic predictor independent of other clinicopathologic factors.

The precise molecular mechanisms for the altered expression of miR-449 in tumors remain unknown. The miR-34 family that shares the same seed sequence with miR-449 is tumor-suppressive and methylated in lung cancer [24], [25], [26]. Moreover, the expression of miR-449 is inactivated by histone H3lys27 (H3K27mes) and reversed by epigenetic drugs for histone modifications [16]. Taken together, aberrant epigenetic events may be important in the mechanisms behind miR-449 dysregulation. MiR-449a is located in the first intron of CDC20B on chromosome 5q11 which is often a site of abnormality in lung cancer [27], [28]. The function of miR-449 in cancer is further supported by experiments in several cancer cell lines in which miR-449 induces G1 arrest, apoptosis, and senescence by regulation of a series of key factors in cell cycle and apoptosis [14], [16], [17], [18], [19]. These results indicate that miR-449 regulates tumor growth as a tumor suppressor, which could partially explain the correlation between low expression of miR-449 and poor prognosis.

Previous studies focused on the role of miR-449 in cell cycle regulation, in this study we found that low expression of miR-449a was correlated with lymph node metastasis. Low expression was also observed in cell lines with higher metastatic potential. These data indicated that, as a potential tumor suppressor, miR-449 might be significant not only in the cell cycle but also in invasion and tumor metastasis by targeting multiple oncogenes. Transwell assays were performed in NSCLC cell lines and the results indicated that miR-449a regulated cell invasion and migration as a tumor suppressor. Of hundreds of potential target genes for miR-449a predicted by three mainstream algorithms, the oncogene c-Met, predicted by all three algorithms, was selected for study. Most recently, c-Met has already been identified as target of miR-449a by luciferase assay in HEK293 cells [29]. Our result agree with the observation that c-Met is a direct target of miR-449a. We demonstrated an inverse correlation between miR-449a and c-Met in NSCLC tissue specimens. *In vitro* overexpression of miR-449a significantly decreased c-Met mRNA and protein expression compared to controls, whereas inhibition of miR-449a resulted in increase in c-Met mRNA and protein. Furthermore, fluorescent reporter assays demonstrated that miR-449a directly bound to the c-Met 3**′**-UTR region.

The cell surface receptor tyrosine kinase c-Met is upregulated in a variety of tumors, including NSCLC [30], [31], [32]. C-Met is important in cell migration and invasion [33], [34], [35], and correlates with prognostic parameters and poor survival in NSCLC [36], [37]. Increased c-Met signaling promotes cell migration and invasion through several pathways such as the focal adhesion kinase (FAK), phosphatidyl inositol 3-kinase (PI3K), and extracellular signal-regulated kinase (ERK) pathways [38], [39]. By directly targeting the gene c-Met, miR-449a inhibited c-Met expression and downstream MMP2 and MMP9, and inhibited cell migration and invasion. Our data provide the first insights into the function of miR-449a in regulating cell migration and invasion in NSCLC cells, at least partially through c-Met.

Currently single miRNAs with multiple functions are potential candidates for gene therapy. Compared with siRNAs, miRNAs have many advantages as novel therapeutic targets such as *in vivo* stability and low toxicity. HDAC1 is a target of miR-449 [12], a recent study showed that in lung cancer, miR-449 has a synergistic effect on growth arrest with HDAC inhibitor [15], providing new information about combination therapy with miRNA and c-Met inhibitors. These results suggested that miR-449 is a potential candidate for miRNA-based therapeutic interventions.

In conclusion, miR-449a, a significantly downregulated miRNA in NSCLC, was associated with lymph node metastasis and poor prognosis. We demonstrated that miR-449a played a crucial role in regulating migration and invasion by targeting c-Met. Although the precise molecular mechanisms require further study, miR-449a provides new insights into prognostic diagnosis and therapeutic strategies for patients with lung cancer.

## Supporting Information

Figure S1 **Relative expression of miR-449a was increased by transfection with an miR-449a mimic.**
(TIF)

Figure S2 **The predicted binding sites of c-Met 3′UTR and miR-449a.**
(TIF)

Table S1 **Clinicopathologic characteristics and follow-up data of 70 FFPET samples from lung cancer patients.**
(DOC)
