# Supplementary figures and images for: MicroRNA-449a Is Downregulated in Non-Small Cell Lung Cancer and Inhibits Migration and Invasion by Targeting c-Met
Source: PLoS One. 2013 May 29;8(5):e64759. doi: 10.1371/journal.pone.0064759 (PMC3667122; doi:10.1371/journal.pone.0064759)

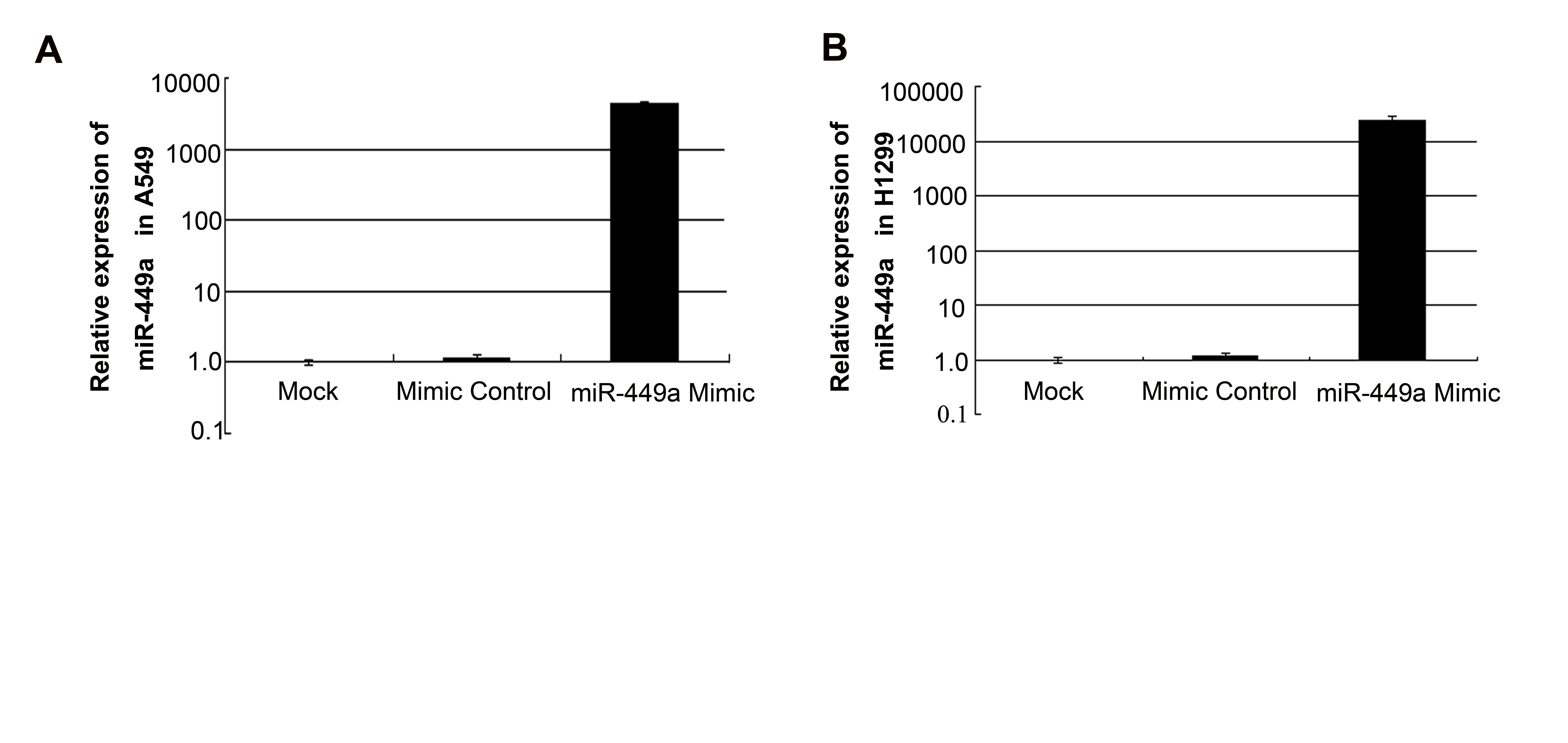

Supplement: Figure S1 — Relative expression of miR-449a was increased by transfection with an miR-449a mimic. (TIF) [file pone.0064759.s001.tif]

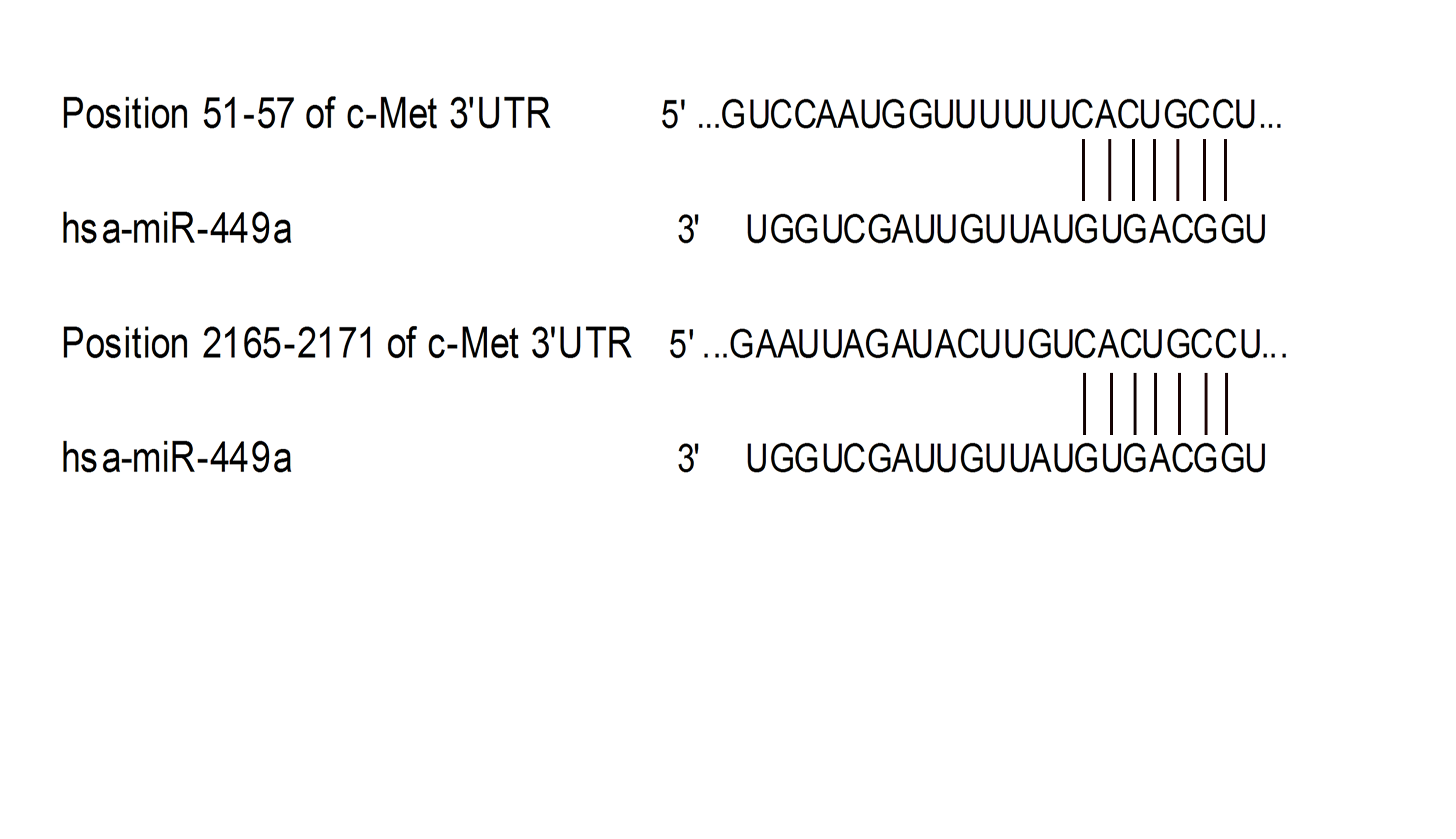

Supplement: Figure S2 — The predicted binding sites of c-Met 3′UTR and miR-449a. (TIF) [file pone.0064759.s002.tif]
