# Supplementary material for: MicroRNA-449a Is Downregulated in Non-Small Cell Lung Cancer and Inhibits Migration and Invasion by Targeting c-Met
Source: PLoS One. 2013 May 29;8(5):e64759. doi: 10.1371/journal.pone.0064759 (PMC3667122; doi:10.1371/journal.pone.0064759)
Supplement: Table S1 — Clinicopathologic characteristics and follow-up data of 70 FFPET samples from lung cancer patients. (DOC) [file pone.0064759.s003.doc]

Table S1

Clinicopathologic characteristics and follow-up data of 70 FFPET samples from lung cancer patients

| Characteristics | number of patients/number analyzed |
| --- | --- |
| Age(median, range) | 56（21-73）(years) |
| Histology type |  |
| Adenocarcinoma | 34/70 |
| Squamous cancer | 36/70 |
| Histological grade |  |
| Ⅰ | 20/70 |
| Ⅱ | 29/70 |
| Ⅲ | 21/70 |
| Pathological stage |  |
| Ⅰ | 21/70 |
| Ⅱ | 28/70 |
| Ⅲ | 21/70 |
| Lymph nodes |  |
| Metastasis | 28/70 |
| No metastasis | 42/70 |
| Overrall survival(median,range) | 36(2-84) (months) |
